# Role of Caveolin-1 in Atrial Fibrillation as an Anti-Fibrotic Signaling Molecule in Human Atrial Fibroblasts

**DOI:** 10.1371/journal.pone.0085144

**Published:** 2014-01-14

**Authors:** Shao-lei Yi, Xiao-jun Liu, Jing-quan Zhong, Yun Zhang

**Affiliations:** 1 Key Laboratory of cardiovascular remodeling and Function Research, Chinese Ministry of Education and Chinese Ministry of Public Health, Department of Cardiology, Qilu Hospital of Shandong University, Jinan, Shandong Province, China; 2 School of Medicine, Shandong University, Jinan, Shandong Province, China; University of Illinois at Chicago, United States of America

## Abstract

Atrial fibrillation (AF) is the most common sustained cardiac arrhythmia in the general population; yet, the precise mechanisms resulting in AF are not fully understood. Caveolin-1 (Cav-1), the principal structural component of caveolae organelles in cardiac fibroblasts, is involved in several cardiovascular conditions; however, the study on its function in atrium, in particular, in AF, is still lacking. This report examines the hypothesis that Cav-1 confers an anti-AF effect by mediating atrial structural remodeling through its anti-fibrotic action. We evaluated the expression of Cav-1, transforming growth factor-β1 (TGF-β1), and fibrosis in atrial specimens of 13 patients with AF and 10 subjects with sinus rhythm, and found that the expression of Cav-1 was significantly downregulated, whereas TGF-β1 level, collagens I/III contents and atrial fibrosis were markedly increased, in AF. Western blot analysis demonstrated that treatment of human atrial fibroblasts (HAFs) with TGF-β1 resulted in a concentration- and time-dependent repression of Cav-1. Downregulation of Cav-1 with siRNA increased the TGF-β1-induced activation of Smad signal pathway and collagens production in HAFs. Furthermore, incubation of HAFs with the peptides derived from Cav-1 to achieve Cav-1 gain-of-function abolished the TGF-β1-induced production of collagens I/III and decreases of MMP-2/-9 expression. Therefore it was concluded that Cav-1 is an important anti-AF signaling mediator by conferring its anti-fibrotic effects in atrium.

## Introduction

Caveolae are 50- to 100-nm omega-shaped invaginations of the cytoplasmic membrane, which were first reported as early as the middle of the last century [1]. Over the past decade, the study on caveolae has blossomed into a rapidly expanding field, and caveolae have been identified as an important member participating in the transcytosis of macromolecules, cholesterol transport and signal transduction in various types of cells [2]–[5]. Caveolin-1 is the first member of the caveolae gene family consisting of three structurally related proteins: caveolin-1 (Cav-1), caveolin-2 (Cav-2), and caveolin-3 (Cav-3) [6], [7]. Cav-1 is the principal structural component of caveolae organelles cells [5], [8]. In the cardiovascular system, Cav-1 and Cav-2 are co-expressed in a variety of cells and tissue types but are most abundantly present in fibroblasts and endothelial cells, whereas Cav-3 is strictly expressed in cardiomyocytes [6], [7], [9].

Atrial fibrillation(AF),the most common cardiac arrhythmia, is frequently accompanied by atrial interstitial fibrosis. A plethora of studies in animal models of atrial fibrillation (AF) and clinical AF have verified that AF is associated with progressive atrial structural and electrical remodeling [11], [12]. The consequence of atrial structural remodeling leading to atrial fibrosis in the development of AF has been demonstrated in many studies [12]. Indeed, enhanced atrial fibrosis markedly increased AF susceptibility [11]. During the development of cardiac fibrosis, the transforming growth factor-β1 (TGF-β1) is considered to be the key profibrotic cytokine [13]. Verheule et al [14] have reported that active TGF-β1 promoted atrial interstitial fibrosis, which was shown to correspond to an increase in atrial conduction heterogeneity and AF vulnerability. In the heart, it was verified that Cav-1 was an inhibitor of the TGF-β1 signaling pathway. Cav-1-knockout animals displayed enhanced TGF-β1 signaling activities, as reflected by more widespread collagen deposition accompanied by reduced expression of matrix metalloproteinases MMP-8 and MMP-13 mRNAs in the heart [15]. This would imply that Cav-1 can cause subordinate alterations in cardiac structure and function by regulating cardiac fibrosis.

In view of all these findings, we hypothesized that Cav-1 might confer an anti-AF effect by participating in the atrial structural remodeling process through its anti-fibrotic action. The carboxyl tail of Cav-1, or the Cav-1 scaffolding domain (CSD; residues 82–101 in Cav-1), is the primary structure that interacts with other molecules [8]. CSD-derived peptides are able to elicit the same cellular functions as Cav-1, which is fully cell permeable and has been widely used as a mimic of the full-length Cav-1 in studies of variety cellular functions associated with Cav-1, indicating the peptide is a superior gain-of-function tool for studying the function of Cav-1 [16]–[19]. Therefore, basing on results of our research on changes in the atrial tissue of AF, an in vitro study was conducted to examine our hypothesis whether Cav-1 could reverse the pathological atrial structural remodeling in patients with AF by using a gain-of-function approach with the CSD peptide. The results provided strong experimental evidence in support of our hypothesis.

## Materials and Methods

### Patients

This study was approved by Qi Lu Hospital Committee of Shan Dong University for Human. Patients (n = 23) consisted of 15 females and 9 males (mean age of 52.58±11.0; range 29–71 years) presenting with rheumatic heart disease and undergoing mitral/aortic valve replacement between January 11, 2012 and June 30, 2012. Thirteen were diagnosed with AF (AF group) and 10 with no history of AF (sinus rhythm [SR] group) (Table 1). None of the study subjects had hyperthyroidism, hypertension, coronary heart disease, dilated cardiomyopathy, diabetes, chronic pulmonary heart disease, or had taken ACE inhibitors or angiotensin receptor blockers recently (5 half-lives of the drug). After written informed consent was obtained, atrial tissue samples from the right atrial appendage were obtained from all subjects. The samples were then used for primary culturing of human atrial fibroblasts, or frozen and stored at −80°C for RNA and protein extraction for qRT-PCR and Western blot, respectively, or fixed by 10% neutralized formalin for histopathology analysis, as described in the File S1. This study conformed to the principles outlined in the Declaration of Helsinki, 1997.

**Table 1:** Patients' Characteristics.

	SR(n = 10)	AF(n = 13)
**Age(years)**	**53.2±13.4**	**52.7±9.7**
**Sex**	**4M/6F**	**5M/8F**
**EF (%)**	**58.9±7.2**	**58.8±8.0**
**LA diameter(mm)**	**47.8±4.9**	59.4±10.5*
**Cardiac Function(NYHA)**	**2II/8 III**	**3II/10 III**

There were no significant statistical differences between sinus rhythm group and AF group in sex, the kind of EF and degrees of cardiac function. *P<0.01 vs. SR. Atrial fibrillation, atrial fibrillation, SR, sinus rhythm; EF, left ventricular ejection fraction; LA, left atrium, NYHA, the New York Heart Association.

### Reagents

Cell culture reagents were purchased from Gibco-BRL (Grand Island, NY, USA). Recombinant human TGF-β1 was obtained from Peprotech (Rehovot, Israel). The anti-Cav-1 antibody was purchased from Cell Signaling Technology (Danvers, MA, USA). Antibodies specific to collagen I, collagen III, TGF-β1, and β-actin were purchased from Abcam (Hong Kong, China). The antibodies to phosphorylated Smad 2, phosphorylated Smad 3, and total Smad 2/3 were purchased from Santa Cruz Biotechnology (Santa Cruz, CA USA). Horseradish peroxidase conjugated immunoglobulin (IgG) was purchased from Jinqiao (Zhongshan, China).

The CSD peptide (amino acids 82–101 of Cav-1; DGIWKASFTTFTVTKYWFYR) and a scrambled control peptide (WGIDKAFFTTSTVTYKWFRY) were synthesized as fusion peptides to the COOH terminus of the antennapedia internalization sequence (RQIKIWFQNRRMKWKK). Before each experiment, lyophilized peptides were dissolved to a final concentration of 1 mM in 10% DMSO as described by Bernatchez et al. [19].

### Cell culture

Human atrial fibroblasts (HAFs) were derived from biopsies of the right atrial appendage, as previous described [20], [21]. The biopsies were obtained from patients with sinus rhythm undergoing mitral/aortic valve replacement. Experiments were performed on cells from up to 9 patients. Cells derived from different subjects were incubated and treated separately. The same passage cells derived from the same subject were considered as a batch. Cells in the same batch were parallel processed. The results were expressed as the fold changes compared to the control group in the same batch, in order to avoid the affect of variability in different subjects. Cells passaged 2–4 times were plated in 6-well plates and allowed to grow to 80% confluency on gelatin-coated wells (6-well plates) at 37°C in a humidified atmosphere of 5% CO2/95% air for 24 h in Dulbecco's Modified Eagle's Medium (4∶1) containing 10% fetal bovine serum. Serum was withdrawn 24 h before incubation with 100 ng/ml recombinant human TGF-β1 for 0, 6, 12, 24 and 48 h, or with TGF-β1 (0, 0.1, 1, 10, 100 and 1000 ng/ml) for 48 h. HAFs were also pretreated for 30 min with the CSD peptide (5 µM) or the scrambled peptide (Scr peptide; 5 µM), and subsequently incubated for 48 h with or without 100 ng/ml TGF-β1. Cells were then harvested from each experimental group for Western blot analysis or qRT-PCR, as described in the File S1.

### siRNA transfection

HAFs were plated in 6-well plates with 80% final density, and transfected with caveolin-1 siRNA (sense 5′-GCCGUGUCUAUUCCA UCUA-3′; antisense 5′-UAGAUGGAAU AGACACGGC-3′) or a non-silencing negative control siRNA (sense 5′-UUCUCCGAACGUGU CACGU-3′; antisense 5′-ACGUGACACGUUC GGAGAA-3′) using Hiperfect transfection reagent (Introgen) following protocols provided by the manufacturer. After 48 h of incubation the cells were either harvested for protein extraction or treated with TGF-β1 as described above.

### Statistical analysis

Data are expressed as the mean ± SE. Statistical differences between groups were determined using the one-way ANOVA followed by Fisher's protected least significant difference (Fisher's PLSD) test. The statistical tests were two-tailed, with a p-value of <0.05 for significance. The statistical analysis was performed with SPSS 16.0 software (SPSS, Chicago, IL).

## Results

### Downregulation of atrial Cav-1 and increase of atrial fibrosis in AF patients

There were no significant differences between the SR and AF group in sex, age, types of valve disease, ejection fraction and degrees of cardiac function. Left atrial diameter in AF group was larger than the atrial diameter observed the SR group (59.4±10.5 vs. 47.8±4.9 *P* = 0.004) (Table 1).

**Figure 1:**
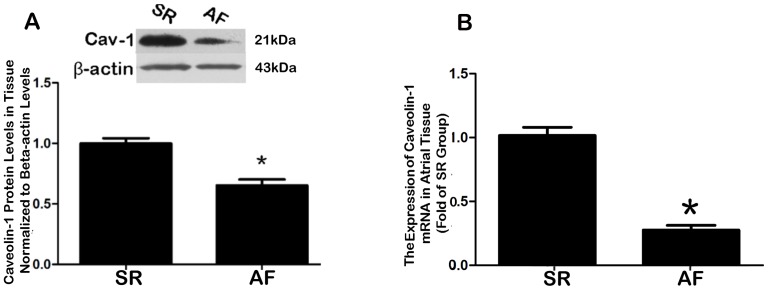
Quantitative analyses of caveolin-1 (Cav-1) expression in human atrial tissues. **A**, Comparison of atrial Cav-1 protein levels between AF and SR subjects, as determined by Western blot analysis. The left panel shows the representative bands, and the right panel the shows the mean data of band density normalized to β-actin. *P<0.001 vs. SR, n = 10; **B**, Comparison of atrial Cav-1 mRNA levels between AF and SR subjects, as determined by real-time RT-PCR (qRT-PCR). *P<0.001 vs. SR; n = 10. Note that the expression of Cav-1 was decreased at both protein and transcript levels in patients with AF compared to patients with SR. Cav-1, caveolin-1; SR, sinus rhythm; AF, atrial fibrillation.

qRT-PCR analyses were used to compare the gene expression of Cav-1 Cav-2 and Cav-3 in atrial tissues of AF and SR patients. No significant differences were observed for Cav-2 and Cav-3 between SR and AF groups (Fig. S1 and S2). However, the Cav-1 mRNA was downregulated in AF relative to SR (Fig. 1B; P<0.001). The result of western blot consisted with qRT-PCR analyses and showed that the protein level of Cav-1 was approximately 30% lower in AF subjects than in the SR group (Fig. 1A, P<0.001).

**Figure 2:**
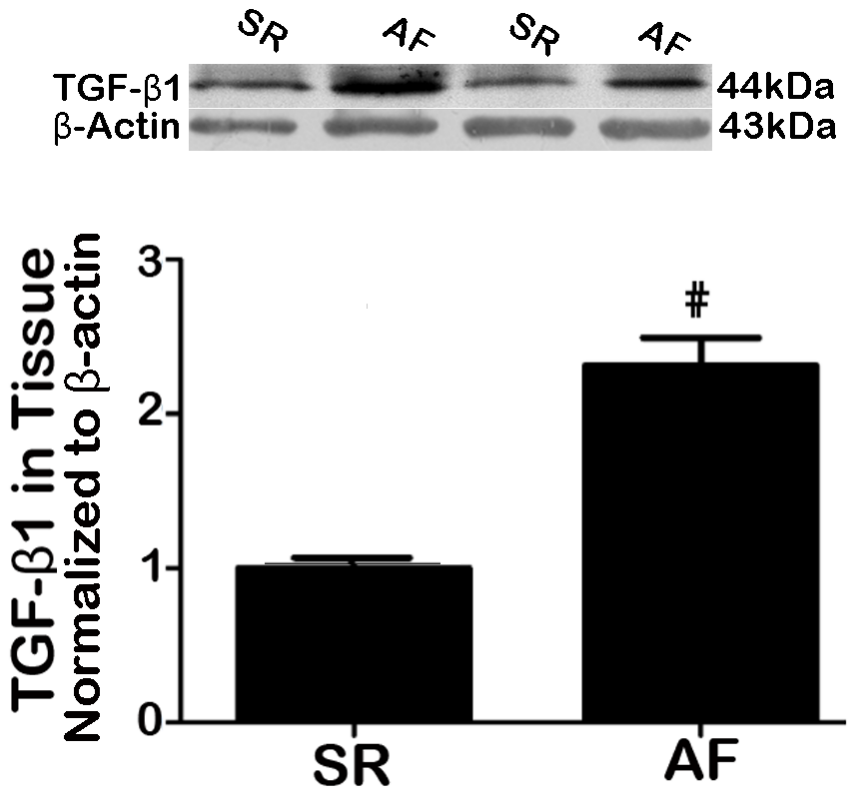
Comparison of TGF-β1 protein levels between AF and SR tissues. Upper panel: representative analog data of Western blot bands; lower panel: averaged analog data of Western blot bands (band density), normalized to β-actin. #P = 0.00 vs. SR, †P = 0.02 vs. SR; n = 10.

**Figure 3:**
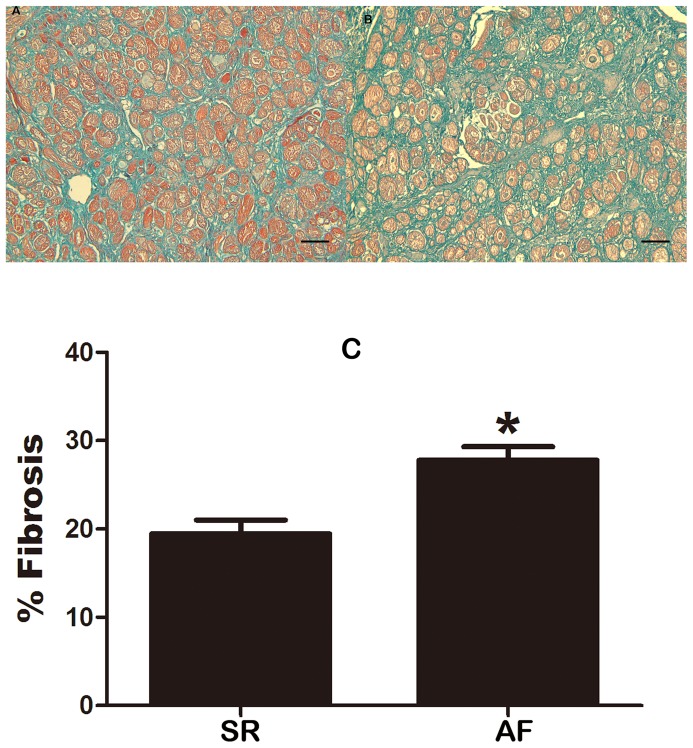
Comparison of atrial fibrosis between AF and SR subjects. **A**, Masson-Trichrome staining sections showing the distribution of fibrosis in AF. **B**, Masson-Trichrome staining sections showing the distribution of fibrosis in SR. **C**, Percentage of fibrotic tissues quantified based on the Masson-Trichrome staining using Image-ProPlus. Note that the proportion of fibrosis was considerably greater in AF subjects than in SR subjects. SR, sinus rhythm; AF, atrial fibrillation. * P<0.001 vs. SR; n = 10. Scale bar = 100 µm. Magnification: ×200.

**Figure 4:**
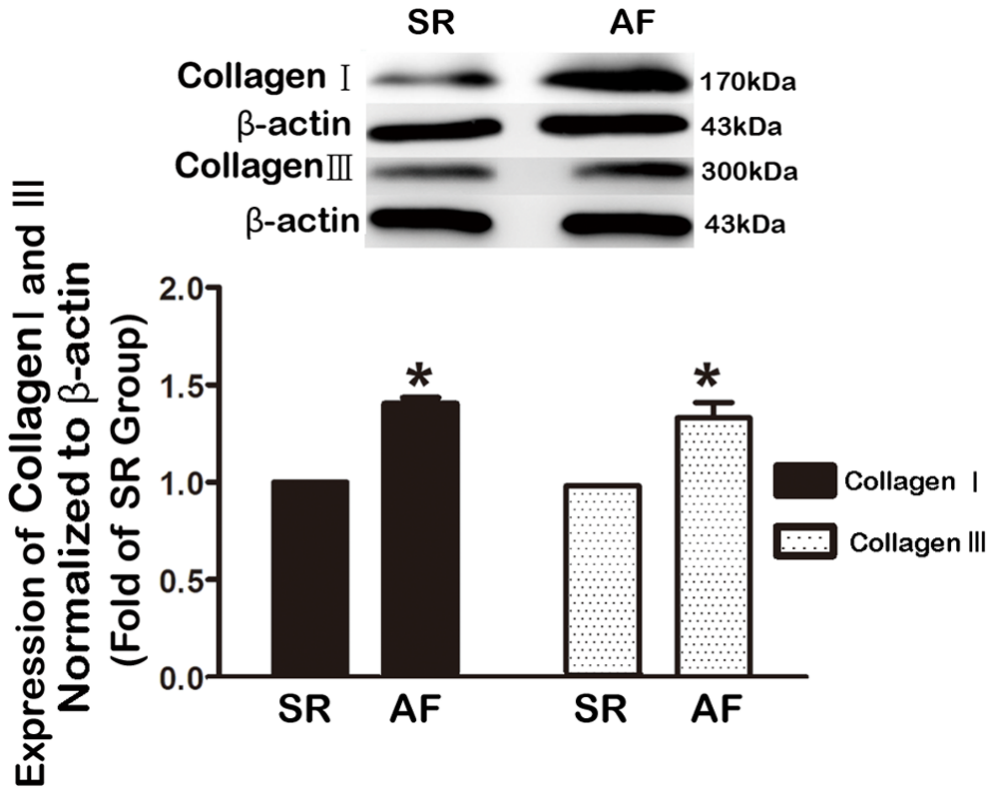
Comparison of atrial contents of collagen I and collagen III in atrial tissues from AF and SR subjects. Collagen contents were determined by Western blot analysis. Left panel: Typical examples of Western blot bands showing upregulation of both collagen I and collagen III; right panel: averaged band densities, normalized to β-actin. Note that the collagen contents were substantially higher in atrial tissues from AF patients than from SR controls. SR, sinus rhythm; AF, atrial fibrillation. *P<0.05; n = 10.

The protein level of transforming growth factor beta 1 (TGF-β1), a key profibrotic cytokine [22], [23], was significantly elevated (∼2.5 fold) in AF patients relative to SR subjects (Fig. 2; P<0.001). And a negative correlation between TGF-β1 and Cav-1 levels was identified (r = 0.75, P = 0.012). In addition, there was a much higher percentage of fibrotic tissues (defined by the significant area stained with Masson-trichrome) in AF than in SR tissues (Fig. 3). Furthermore, the level of collagen I, the main component of the extracellular matrix in cardiac fibrosis, and collagen III were markedly greater in atrial tissues from AF patients than from SR subjects (Fig. 4). These results clearly indicated that there was a higher degree of fibrosis in AF patients.

### Downregulation of Cav-1 by TGF-β1 in human atrial fibroblasts (HAFs)

**Figure 5:**
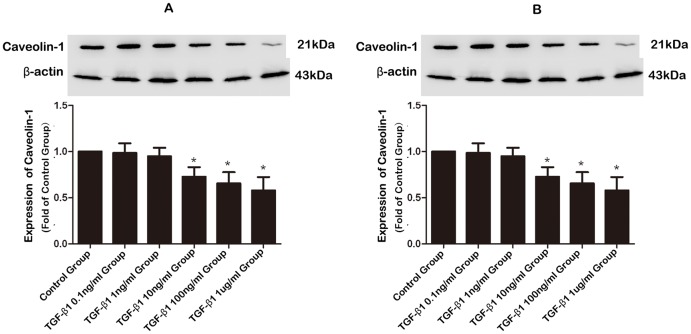
Effects of TGF-β1 on the expression of Cav-1 in human atrial fibroblasts (HAF). **A**, Time-dependent alterations of Cav-1 protein levels assessed by Western blotting in HAFs incubated with TGF-β1 of 100 ng/ml for varying periods (0, 6, 12, 24, and 48 h). Note that TGF-β1 induced bi-phasic changes of Cav-1 protein expression with initial upregulation followed by subsequent downregulation. Control HAFs were mock-treated. **B**, Concentration-dependent effects of TGF-β1 on Cav-1 protein levels assessed by Western blot analysis in HAFs exposed to varying concentrations of TGF-β1 (0, 0.1, 1, 10, 100, and 1000 ng/ml) for 48 h. *P<0.05 vs. Control; n = 3 per group with each measurement conducted in triplicate.

To determine if the observed downregulation of Cav-1 was caused by upregulation of TGF-β1, we investigated the effects of exogenous TGF-β1 on expression of Cav-1 in HAFs. To this end, HAFs were incubated with TGF-β1 of a fixed concentration of 100 ng/ml (Fig. 5A) for varying periods (6, 12, 24, or 48 h) or of varying concentrations (0.1, 1, 10, 100, and 1000 ng/ml) for a fixed period of 48 h (Fig. 5B). As illustrated in Figure 5A, bi-phasic time-dependent effects were observed, characterized by an initial upregulation followed by a downregulation. For the first 12 h, TGF-β1 increased Cav-1 protein level by around 35% (P = 0.002); thereafter, however, Cav-1 began to decline and at 48 h, it was reduced by 42% (P = 0.004). Concentration-dependent downregulation of Cav-1 expression by TGF-β1 was also evident, as shown in Figure 5B. Since exposure to 100 ng/ml TGF-β1 for 48 h caused significant downregulation of Cav-1 without inducing cytotoxicity to affect HAF survival as assessed using the MTT assay (data not shown), all subsequent Western blot experiments were performed by incubating cells with TGF-β1 at 100 ng/ml for 48 h.

### Enhanced TGF-β1 induced- Smad signal pathway and collagen production by knock-down caveolin-1 with siRNA in HAFs

**Figure 6:**
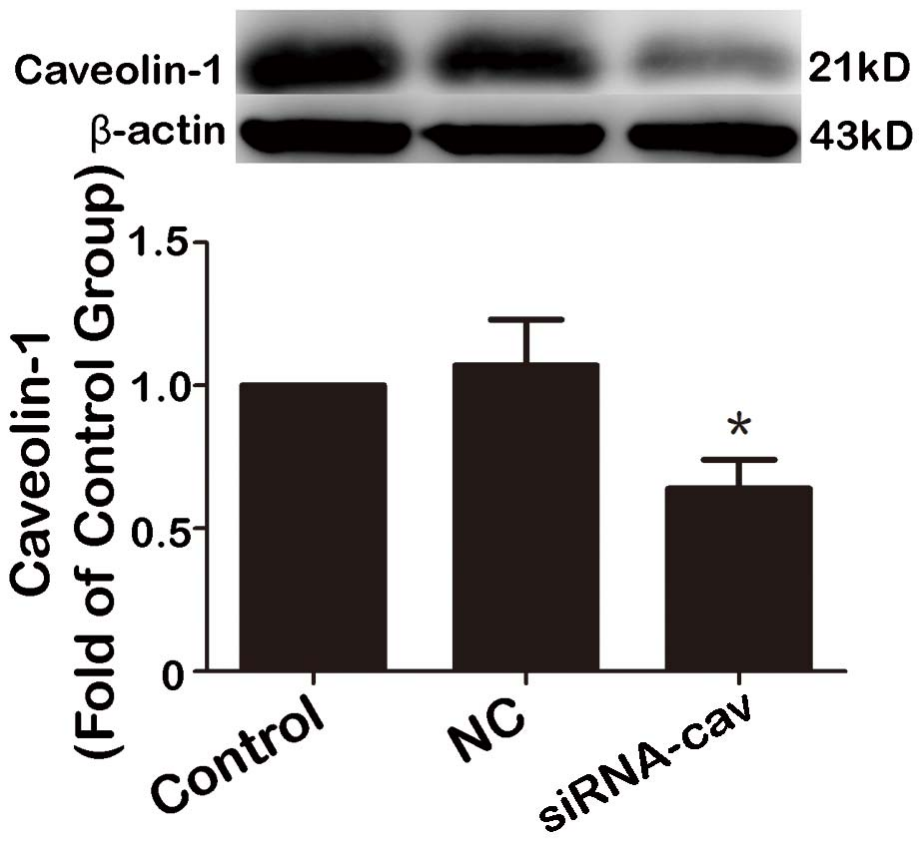
The siRNA of caveolin-1 suppressed the expression of caveolin-1 in HAFs. HAFs were transfected with caveolin-1 siRNA or a non-silencing negative control siRNA using Hiperfect transfection reagent following protocols provided by the manufacturer. After 48 h of incubation the cells were harvested for protein extraction. Not that the caveolin-1 siRNA suppressed the protein level of caveolin-1 by 46% compared to control group. HAFs, human atrial fibroblasts. NC, negative control, siRNA-Cav, the caveolin-1 siRNA. *P = 0.035 vs. control group. n = 3 per group; measurement in triplicate for each single experiment.

**Figure 7:**
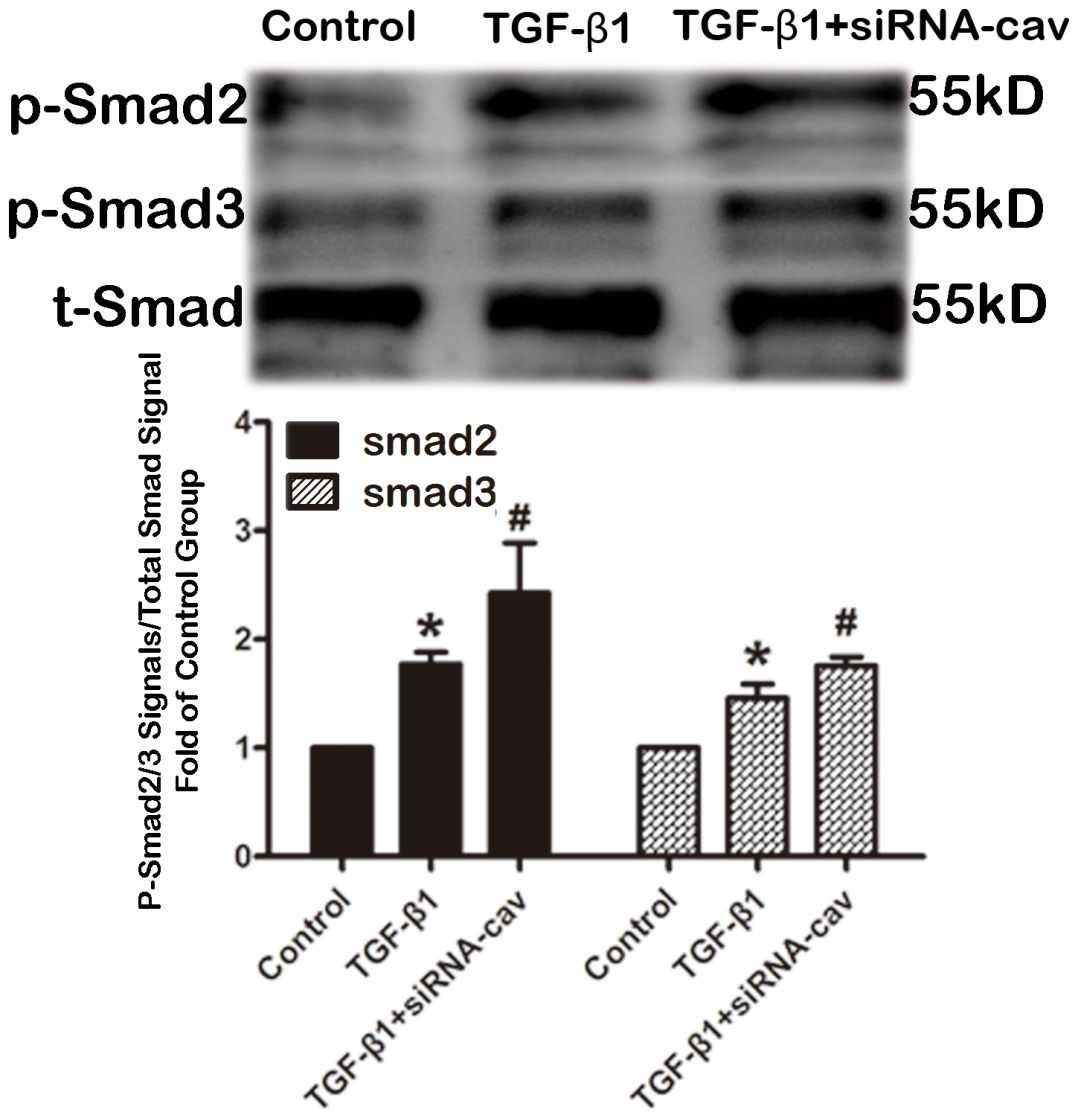
Downregulation of caveolin-1 increased the TGF-β1-induced phosphorylated Smad 2/3. HAFs in the siRNA-Cav group were transfected with the caveolin-1 siRNA using Hiperfect transfection reagent following protocols provided by the manufacturer, and then HAFs were treated with 100 ng TGF-β1 for 48 h. HAFs in TGF-β1 group were only treated with 100 ng TGF-β1 for 48 h. Note that downregulation of caveolin-1 with the caveolin-1 siRNA increased the amount of phosphorylated Smad 2/3 compared to the TGF-β1 group. HAFs, human atrial fibroblasts. siRNA-Cav, the caveolin-1 siRNA. p-Smad2, phosphorylated Smad 2. p-Smad3, phosphorylated Smad 3. t-Smad, the total Smad 2/3. *P<0.05 vs. control group. #P<0.05 vs. TGF-β1 group. n = 3 per group; measurement in triplicate for each single experiment.

**Figure 8:**
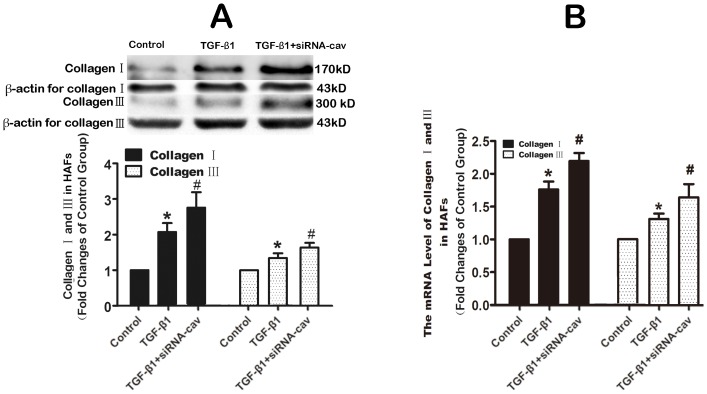
Downregulation of caveolin-1 increased the TGF-β1-induced collagens production. HAFs in the siRNA-cav group were transfected with the caveolin-1 siRNA using Hiperfect transfection reagent following protocols provided by the manufacturer, and then HAFs were treated with 100 ng TGF-β1 for 48 h. HAFs in TGF-β1 group were only treated with 100 ng TGF-β1 for 48 h for western blot or 24 h for qRT-PCR. Note that downregulation of caveolin-1 with the caveolin-1 siRNA increased the TGF-β1-induced the mRNA level (B) and protein level (A) of collagen I and III production in HAFs. HAFs, human atrial fibroblasts. siRNA-Cav, the caveolin-1 siRNA. *P<0.05 vs. control group. #P<0.05 vs. TGF-β1 group. n = 3 per group; measurement in triplicate for each single experiment.

To investigate the impact of downregulating Cav-1 in HAFs, Cav-1 siRNA was used to knock down Cav-1. Cav-1 siRNA suppressed Cav-1 expression by 46% (Fig. 6) and Cav-1 downregulation subsequently increased the amount of Smad2/3 phosphorylation and collagen expression by HAFs (Fig. 7 and 8).

### Inhibition of TGF-β1 induced-collagen production and the activation of Smad signal pathway by the CSD peptide in HAFs

**Figure 9:**
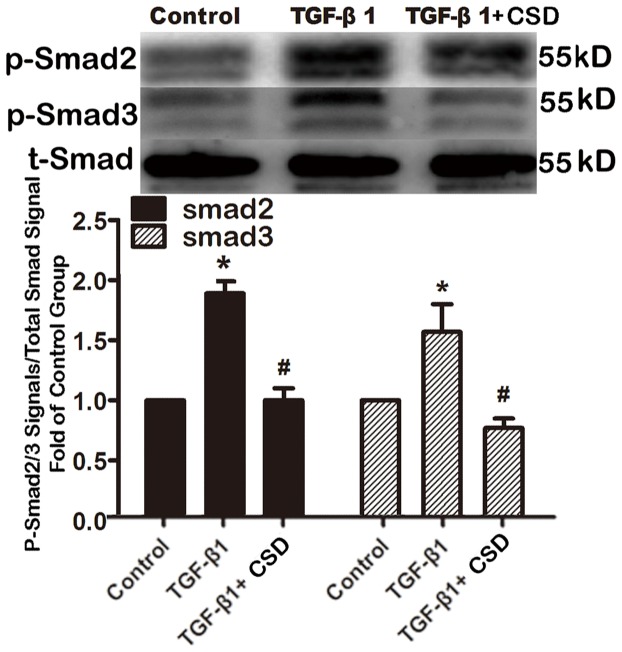
The CSD peptide inhibited TGF-β1-induced activation of Smad 2/3 in HAFs. HAFs were pretreated with the CSD peptide at a concentration of 5 µM or the Scr peptide (5 µM) for 30 min, and subsequently incubated with or without 100 ng/ml TGF-β1 for 48 h. Note that the CSD peptide abrogated the TGF-β1-induced upregulation of phosphorylated Smad 2 and 3. HAFs, human atrial fibroblasts. The CSD peptide, peptides derived from the scaffolding domain of Cav-1. Scr peptide, scrambled peptide. p-Smad2,phosphorylated Smad 2. p-Smad3,phosphorylated Smad 3. t-Smad, the total Smad 2/3. *P<0.05 vs. Control; #P<0.05 vs. TGF-β1; n = 4 per group; measurement in triplicate for each single experiment.

**Figure 10:**
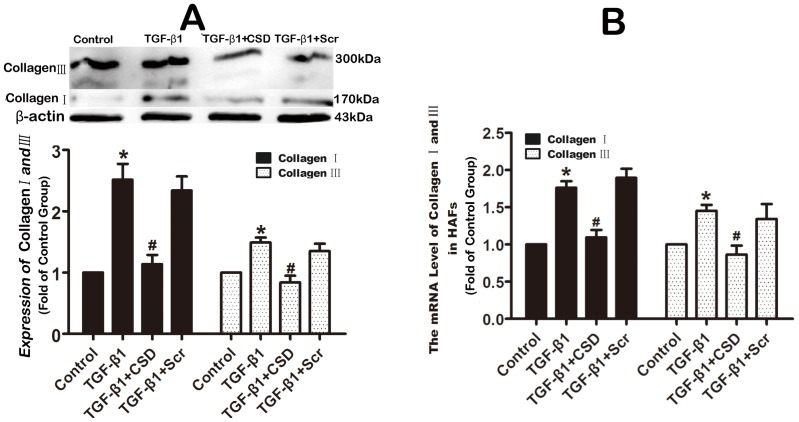
The CSD peptide inhibited TGF-β1-induced upregulation of collagens in HAFs. HAFs were pretreated with the CSD peptide at a concentration of 5 µM or the Scr peptide (5 µM) for 30 min, and subsequently incubated with or without 100 ng/ml TGF-β1 for 48 h for western blot or 24 h for qRT-PCR. Note that the CSD peptide pronouncedly abrogated the TGF-β1-induced increased protein level (A) and mRNA level (B) of collagens. HAFs, human atrial fibroblasts. The CSD peptide, peptides derived from the scaffolding domain of Cav-1. Scr peptide, scrambled peptide. *P<0.05 vs. Control; #P<0.05 vs. TGF-β1; n = 4 per group; measurement in triplicate for each single experiment.

The results presented above suggested that Cav-1 might be involved in modulation of atrial fibrogenesis by participating in the TGF-β1 profibrotic signaling pathway. To examine this notion, HAFs were pretreated with the CSD peptide (which mimics functional Cav-1) at a concentration of 5 µM for 30 min, and subsequently incubated with 100 ng/ml TGF-β1 for 48 hours. Then the collagen content was measured by Western blot. As illustrated in Figure 9 and 10, the CSD peptide inhibited the TGF-β1-induced activation of Smad 2/3 and pronouncedly reduced the TGF-β1-induced increase in production of collagens by 98%. By comparison, the Scr peptide (5 µM), as a negative control, did not alter the effects of TGF-β1.

**Figure 11:**
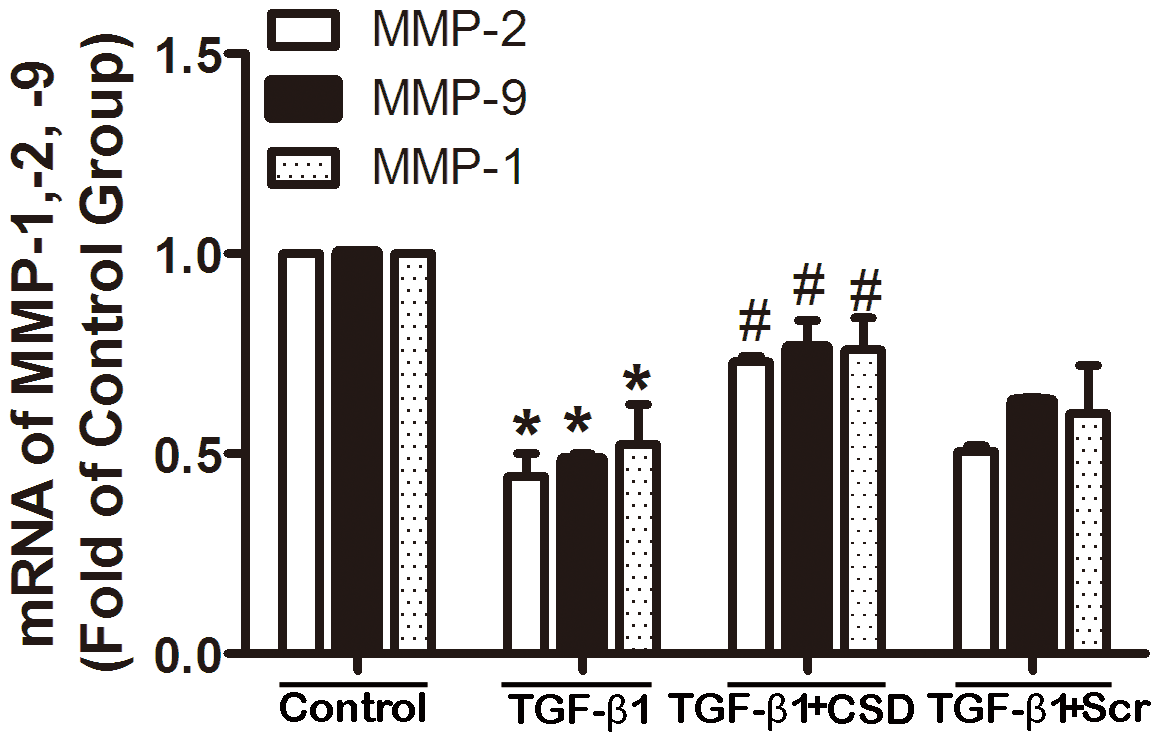
The CSD peptide attenuated TGF-β1-induced downregulation of MMPs in HAFs. HAFs were pretreated with the CSD peptide (5 µM) or the Scr peptide (5 µM) for 30 min, and subsequently incubated with or without 100 ng/ml TGF-β1 for 48 h. Note that the CSD peptide remarkably reversed the TGF-β1-induced downregulation of MMP-1, MMP-2 and MMP-9. MMP, matrix metalloproteinase. HAFs, human atrial fibroblasts. The CSD peptide, peptides derived from the scaffolding domain of caveolin-1; Scr peptide, scrambled peptide. *P<0.05 vs. Control; #P<0.05 vs. TGF-β1. n = 3 per group with each measurement conducted in triplicate.

To evaluate the degradation of collagen, we assessed the effects of the CSD peptide on expression of three principal matrix metalloproteinase collagenases (MMP-1, MMP-2 and MMP-9), which degrade the extracellular matrix [24], [25]. As shown in Figure 11, the CSD peptide (5 µM) attenuated TGF-β1 induced-decrease of MMPs, whereas the Scr peptide did not significantly affect TGF-β1-mediated changes to MMPs expression levels.

## Discussion

The present study examined the expression alteration of Cav-1 and its possible relationship with the TGF-β1 pro-fibrotic signaling pathway in AF patients and human atrial fibroblasts. The main findings of this study included: (1) Cav-1 was significantly downregulated at both transcript and protein levels, which was accompanied by an upregulation of TGF-β1, an increase of collagens content, and stimulation of atrial fibrosis in atrial tissues from AF patients relative to SR subjects; (2) Application of TGF-β1 to HAFs decreased the protein level of Cav-1; (3) Downregulation of Cav-1 in HAFs increased the TGF-β1-induced activation of Smad signal pathway and collagens production. (4) Utilizing the CSD peptide to achieve Cav-1 gain-of-function mitigated the TGF-β1 pro-fibrotic signaling pathway, leading to reduction of collagens content in HAFs. Based upon these findings, it was concluded that Cav-1 is an important anti-AF signaling mediator through its anti-fibrotic effects in atrial tissue.

Cav-1 is the predominant caveolin isoform in the cardiovascular system, and it drives the formation of caveolae, as evidenced by studies depleting Cav-1 wherein loss of Cav-1 results in loss of caveolae [26]. Most of the published studies on Cav-1 and cardiovascular diseases employed Cav-1 deficient models, and researches on the mechanisms responsible for change in Cav-1 expression during these pathological processes are scarce. One study reported a loss of Cav-1 expression in the myocardium during ischemia-reperfusion injury [27]. Another study documented a downregulation of Cav-1 in the heart of dogs with experimental hypertension [28]. In contrast, upregulation of Cav-1 was found at both protein and mRNA levels in the failing human heart [29]. However, to our knowledge, there have no published data regarding Cav-1 expression in atrial tissue; our study therefore represents the first to report the alteration, specifically, downregulation, of Cav-1 expression in atrial tissues of AF patients. This downregulation might be induced by TGF-β1 as the Cav-1 downregulation was in parallel to TGF-β1 upregulation in AF atria, and more notably, exposure of HAFs to TGF-β1 diminished the expression levels of Cav-1 mRNA and protein.

Increasing evidence suggests that Cav-1 acting as a scaffolding protein that functionally regulates signaling molecules plays a crucial role in the pathogenesis of many diseases [30]–[32]. Several studies have suggested a role for Cav-1 in the development of ventricular cardiac hypertrophy [33]. For example, Cav-1 deficient mice have showed cardiac hypertrophy owing to hyperactivation of ERK 1/2 signaling under basal conditions [34]. Similarly, another study found that Cav-1 knock-out mice displayed dilated left ventricles and right ventricular hypertrophy [35]. Furthermore, in mice undergone left anterior descending coronary artery ligation, lacking Cav-1 failed to increase β-adrenergic receptor density, leading to reduced cAMP production, PKA phosphorylation, and survival [36]. Volonte et al [37]demonstrated that Cav-1 and Cav-3 are co-expressed in mouse and rat atrial cardiomyocytes, and that they can interact and form heterooligomeric complexes in atrial cells. In Cav-1 deficient mice, expression of the ATP-gated ion channel P2X7R was increased in atrial cardiomyocytes [38]. Nevertheless, prior to the current study, the roles of Cav-1 in atrial pathophysiology have not been explored. In the present study, provided strong evidence that downregulation of Cav-1 in atrial tissues contributes to increased atrial fibrosis in the setting of AF; this is in accordance with the study showing an increase in right ventricular interstitial fibrosis in Cav-1-/- mice [10].

Atrial interstitial fibrosis is a basic pathological feature of AF [39], [40]. Collagens I and III, the major matrix proteins of cardiocytes, constitute 85% of the content of the myocardial matrix. Increased fibrosis and collagens were often found in atria of AF patients as opposed to those patients with sinus rhythm as shown in the present study. TGF-β1 is a key regulator of fibrosis that enhances collagen synthesis both in vitro and in vivo thereby affecting remodeling of the ECM [41], [42]. Therefore enhancing TGF-β1 signaling activity would result in cardiac fibrosis [43]. According to previous research [44] and our results, Cav-1 is a negative regulator of TGF-β1, and decrease of Cav-1 results in the hyperactivity of TGF-β1 Smad 2/3 signal pathway and an increased collagen production. It was speculated that the reduced expression of Cav-1 in AF patients results in the enhanced activities of the TGF-β1 profibrotic signaling pathway, which promotes the atrial fibrosis in AF. In the present study, the CSD peptides abolished the TGF-β1-induced activation of Smad 2/3 signal pathway and the expression of collagens I/III in HAFs, demonstrating that Cav-1 may be able to inhibit the atrial fibrosis in AF.

Collagen deposition is the result of a balance between the expression of collagen genes and its degradation by MMPs [45]. The primary function of MMPs is to decompose collagens and reduce collagen content, and their proteolytic activities are diminished in AF. It is therefore conceivable that changes of Cav-1 expression and function could have profound impacts on the expression and function of MMPs. Downregulation of Cav-1 in AF revealed in this study may be, at least partially, a cause for reduced MMPs function in AF; this is evidenced by our finding that Cav-1 was able to reverse the downregulation of expression of MMP-1, MMP-2 and MMP-9 induced by TGF-β1 in HAFs. Taken all together, it appears that in AF, TGF-β1 expression and signaling activity are enhanced which results in downregulation of Cav-1. The latter in turn promotes expression and function of MMPs leading to increased collagen production thereby atrial fibrosis. While this notion may be a mechanism to support the observed results in this study, it definitely merits future investigations to acquire more conclusive evidence.

In the present study, only the in vitro experiment was conducted to examine the effect of Cav-1 on the atrial pathological changes in AF, which was the major limitation of the research. Data presented in this report revealed that Cav-1 is an important anti-AF signaling mediator through its anti-fibrotic effects in atrial tissue which might be give us some clues that interventions targeting on Cav-1 could be new strategies for reversing adverse atrial structural remodeling in AF, however, the results could not be directly extrapolated to the vivo study, since the in vivo effect of supra physiological concentration of mediators depended on the cellular milieu, and both TGF-β1 and Cav-1 may be affected greatly by other upstream biomolecules in vivo, such as angiotensin II [46], [47]. So in vivo studies are needed to further explore the role of caveolin-1 in AF.

Probably the most important finding in this study is that application of the CSD peptide to mimic the function of Cav-1 minimized collagen production. Data presented in this report suggested that Cav-1 is an important regulator of AF. However, this study merely lays the groundwork for future studies on the role of Cav-1 in AF. Additional studies are warranted to confirm the results.

## Supporting Information

Figure S1 **Comparison of atrial Cav-2 mRNA levels between AF and SR subjects, as determined by real-time RT-PCR (qRT-PCR).** P = 0.325, n = 6. Note that the gene expression of Cav-2 showed no significant difference between groups. Cav-2, caveolin-2; SR, sinus rhythm; AF, atrial fibrillation.
(TIF)

Figure S2 **Comparison of atrial Cav-3 mRNA levels between AF and SR subjects, as determined by real-time RT-PCR (qRT-PCR).** P = 0.943, n = 4. Note that the gene expression of Cav-3 showed no significant difference between groups. Cav-3, caveolin-3; SR, sinus rhythm; AF, atrial fibrillation.
(TIF)

File S1 **The protocols of western blot, qRT-PCR and Masson Staining.**
(DOC)

## References

[1] Yamada E (1955) The fine structure of the gall bladder epithelium of the mouse. J Biophys Biochem Cytol 1: 445–458. 13263332 10.1083/jcb.1.5.445 PMC2229656

[2] Li S, Okamoto T, Chun M, Sargiacomo M, Casanova JE, et al (1995) Evidence for a regulated interaction between heterotrimeric G proteins and caveolin. J Biol Chem 270: 15693–15701. 7797570 10.1074/jbc.270.26.15693

[3] Fu C, He J, Li C, Shyy JY, Zhu Y (2010) Cholesterol increases adhesion of monocytes to endothelium by moving adhesion molecules out of caveolae. Biochim Biophys Acta 1801: 702–710. 20382259 10.1016/j.bbalip.2010.04.001

[4] Xu Y, Krause A, Hamai H, Harvey BG, Worgall TS, et al (2010) Proinflammatory phenotype and increased caveolin-1 in alveolar macrophages with silenced CFTR mRNA. PLoS One 5: e11004. 20543983 10.1371/journal.pone.0011004 PMC2882373

[5] Harris J, Werling D, Hope JC, Taylor G, Howard CJ (2002) Caveolae and caveolin in immune cells: distribution and functions. Trends Immunol 23: 158–164. 11864845 10.1016/s1471-4906(01)02161-5

[6] Scherer PE, Lewis RY, Volonte D, Engelman JA, Galbiati F, et al (1997) Cell-type and tissue-specific expression of caveolin-2. Caveolins 1 and 2 co-localize and form a stable hetero-oligomeric complex in vivo. J Biol Chem 272: 29337–29346. 9361015 10.1074/jbc.272.46.29337

[7] Tang Z, Scherer PE, Okamoto T, Song K, Chu C, et al (1996) Molecular cloning of caveolin-3, a novel member of the caveolin gene family expressed predominantly in muscle. J Biol Chem 271: 2255–2261. 8567687 10.1074/jbc.271.4.2255

[8] Smart EJ, Graf GA, McNiven MA, Sessa WC, Engelman JA, et al (1999) Caveolins, liquid-ordered domains, and signal transduction. Mol Cell Biol 19: 7289–7304. 10523618 10.1128/mcb.19.11.7289 PMC84723

[9] Cruz JA, Bauer EM, Rodriguez AI, Gangopadhyay A, Zeineh NS, et al (2012) Chronic hypoxia induces right heart failure in caveolin-1-/- mice. Am J Physiol Heart Circ Physiol 302: H2518–2527. 22505641 10.1152/ajpheart.01140.2011 PMC3378264

[10] Pan CH, Lin JL, Lai LP, Chen CL, Stephen Huang SK, et al (2007) Downregulation of angiotensin converting enzyme II is associated with pacing-induced sustained atrial fibrillation. FEBS Lett 581: 526–534. 17254576 10.1016/j.febslet.2007.01.014

[11] Abed HS, Samuel CS, Lau DH, Kelly DJ, Royce SG, et al (2013) Obesity results in progressive atrial structural and electrical remodeling: implications for atrial fibrillation. Heart Rhythm 10: 90–100. 23063864 10.1016/j.hrthm.2012.08.043

[12] Kostin S, Klein G, Szalay Z, Hein S, Bauer EP, et al (2002) Structural correlate of atrial fibrillation in human patients. Cardiovasc Res 54: 361–379. 12062341 10.1016/s0008-6363(02)00273-0

[13] Everett THt, Olgin JE (2007) Atrial fibrosis and the mechanisms of atrial fibrillation. Heart Rhythm 4: S24–27. 17336879 10.1016/j.hrthm.2006.12.040 PMC1850572

[14] Verheule S, Sato T, Everett Tt, Engle SK, Otten D, et al (2004) Increased vulnerability to atrial fibrillation in transgenic mice with selective atrial fibrosis caused by overexpression of TGF-beta1. Circ Res 94: 1458–1465. 15117823 10.1161/01.RES.0000129579.59664.9d PMC2129102

[15] Miyasato SK, Loeffler J, Shohet R, Zhang J, Lindsey M, et al (2011) Caveolin-1 modulates TGF-beta1 signaling in cardiac remodeling. Matrix Biol 30: 318–329. 21641995 10.1016/j.matbio.2011.05.003 PMC4489541

[16] Bucci M, Gratton JP, Rudic RD, Acevedo L, Roviezzo F, et al (2000) In vivo delivery of the caveolin-1 scaffolding domain inhibits nitric oxide synthesis and reduces inflammation. Nat Med 6: 1362–1367. 11100121 10.1038/82176

[17] Oka N, Yamamoto M, Schwencke C, Kawabe J, Ebina T, et al (1997) Caveolin interaction with protein kinase C. Isoenzyme-dependent regulation of kinase activity by the caveolin scaffolding domain peptide. J Biol Chem 272: 33416–33421. 9407137 10.1074/jbc.272.52.33416

[18] Tourkina E, Richard M, Gooz P, Bonner M, Pannu J, et al (2008) Antifibrotic properties of caveolin-1 scaffolding domain in vitro and in vivo. Am J Physiol Lung Cell Mol Physiol 294: L843–861. 18203815 10.1152/ajplung.00295.2007

[19] Bernatchez PN, Bauer PM, Yu J, Prendergast JS, He P, et al (2005) Dissecting the molecular control of endothelial NO synthase by caveolin-1 using cell-permeable peptides. Proc Natl Acad Sci U S A 102: 761–766. 15637154 10.1073/pnas.0407224102 PMC545535

[20] Turner NA, Porter KE, Smith WH, White HL, Ball SG, et al (2003) Chronic beta2-adrenergic receptor stimulation increases proliferation of human cardiac fibroblasts via an autocrine mechanism. Cardiovasc Res 57: 784–792. 12618240 10.1016/s0008-6363(02)00729-0

[21] Porter KE, Turner NA, O'Regan DJ, Ball SG (2004) Tumor necrosis factor alpha induces human atrial myofibroblast proliferation, invasion and MMP-9 secretion: inhibition by simvastatin. Cardiovasc Res 64: 507–515. 15537504 10.1016/j.cardiores.2004.07.020

[22] Khan R, Sheppard R (2006) Fibrosis in heart disease: understanding the role of transforming growth factor-beta in cardiomyopathy, valvular disease and arrhythmia. Immunology 118: 10–24. 16630019 10.1111/j.1365-2567.2006.02336.x PMC1782267

[23] Redondo S, Navarro-Dorado J, Ramajo M, Medina U, Tejerina T (2012) The complex regulation of TGF-beta in cardiovascular disease. Vasc Health Risk Manag 8: 533–539. 23028232 10.2147/VHRM.S28041 PMC3446857

[24] Levick SP, Brower GL (2008) Regulation of matrix metalloproteinases is at the heart of myocardial remodeling. Am J Physiol Heart Circ Physiol 295: H1375–1376. 18757475 10.1152/ajpheart.907.2008 PMC2593525

[25] Hori Y, Kashimoto T, Yonezawa T, Sano N, Saitoh R, et al (2012) Matrix metalloproteinase-2 stimulates collagen-I expression through phosphorylation of focal adhesion kinase in rat cardiac fibroblasts. Am J Physiol Cell Physiol 303: C947–953. 22914642 10.1152/ajpcell.00401.2011

[26] Galbiati F, Volonte D, Engelman JA, Watanabe G, Burk R, et al (1998) Targeted downregulation of caveolin-1 is sufficient to drive cell transformation and hyperactivate the p42/44 MAP kinase cascade. EMBO J 17: 6633–6648. 9822607 10.1093/emboj/17.22.6633 PMC1171009

[27] Chaudhary KR, Cho WJ, Yang F, Samokhvalov V, El-Sikhry HE, et al (2013) Effect of ischemia reperfusion injury and epoxyeicosatrienoic acids on caveolin expression in mouse myocardium. J Cardiovasc Pharmacol 61: 258–263. 23403888 10.1097/FJC.0b013e31827afcee

[28] Piech A, Massart PE, Dessy C, Feron O, Havaux X, et al (2002) Decreased expression of myocardial eNOS and caveolin in dogs with hypertrophic cardiomyopathy. Am J Physiol Heart Circ Physiol 282: H219–231. 11748066 10.1152/ajpheart.2002.282.1.H219

[29] Uray IP, Connelly JH, Frazier OH, Taegtmeyer H, Davies PJ (2003) Mechanical unloading increases caveolin expression in the failing human heart. Cardiovasc Res 59: 57–66. 12829176 10.1016/s0008-6363(03)00352-3

[30] Jin Y, Lee SJ, Minshall RD, Choi AM (2011) Caveolin-1: a critical regulator of lung injury. Am J Physiol Lung Cell Mol Physiol 300: L151–160. 21097526 10.1152/ajplung.00170.2010 PMC4380484

[31] Patani N, Martin LA, Reis-Filho JS, Dowsett M (2012) The role of caveolin-1 in human breast cancer. Breast Cancer Res Treat 131: 1–15. 21901387 10.1007/s10549-011-1751-4

[32] Engel D, Beckers L, Wijnands E, Seijkens T, Lievens D, et al (2011) Caveolin-1 deficiency decreases atherosclerosis by hampering leukocyte influx into the arterial wall and generating a regulatory T-cell response. FASEB J 25: 3838–3848. 21795505 10.1096/fj.11-183350 PMC3672907

[33] Murata T, Lin MI, Huang Y, Yu J, Bauer PM, et al (2007) Reexpression of caveolin-1 in endothelium rescues the vascular, cardiac, and pulmonary defects in global caveolin-1 knockout mice. J Exp Med 204: 2373–2382. 17893196 10.1084/jem.20062340 PMC2118452

[34] Cohen AW, Park DS, Woodman SE, Williams TM, Chandra M, et al (2003) Caveolin-1 null mice develop cardiac hypertrophy with hyperactivation of p42/44 MAP kinase in cardiac fibroblasts. Am J Physiol Cell Physiol 284: C457–474. 12388077 10.1152/ajpcell.00380.2002

[35] Zhao YY, Liu Y, Stan RV, Fan L, Gu Y, et al (2002) Defects in caveolin-1 cause dilated cardiomyopathy and pulmonary hypertension in knockout mice. Proc Natl Acad Sci U S A 99: 11375–11380. 12177436 10.1073/pnas.172360799 PMC123264

[36] Jasmin JF, Rengo G, Lymperopoulos A, Gupta R, Eaton GJ, et al (2011) Caveolin-1 deficiency exacerbates cardiac dysfunction and reduces survival in mice with myocardial infarction. Am J Physiol Heart Circ Physiol 300: H1274–1281. 21297026 10.1152/ajpheart.01173.2010 PMC3075024

[37] Volonte D, McTiernan CF, Drab M, Kasper M, Galbiati F (2008) Caveolin-1 and caveolin-3 form heterooligomeric complexes in atrial cardiac myocytes that are required for doxorubicin-induced apoptosis. Am J Physiol Heart Circ Physiol 294: H392–401. 17982011 10.1152/ajpheart.01039.2007

[38] Barth K, Pfleger C, Linge A, Sim JA, Surprenant A, et al (2010) Increased P2X7R expression in atrial cardiomyocytes of caveolin-1 deficient mice. Histochem Cell Biol 134: 31–38. 20563595 10.1007/s00418-010-0716-8

[39] Gramley F, Lorenzen J, Koellensperger E, Kettering K, Weiss C, et al (2010) Atrial fibrosis and atrial fibrillation: the role of the TGF-beta1 signaling pathway. Int J Cardiol 143: 405–413. 19394095 10.1016/j.ijcard.2009.03.110

[40] Lin CS, Pan CH (2008) Regulatory mechanisms of atrial fibrotic remodeling in atrial fibrillation. Cell Mol Life Sci 65: 1489–1508. 18322650 10.1007/s00018-008-7408-8 PMC11131653

[41] Xiao H, Lei H, Qin S, Ma K, Wang X (2010) TGF-beta1 expression and atrial myocardium fibrosis increase in atrial fibrillation secondary to rheumatic heart disease. Clin Cardiol 33: 149–156. 20235205 10.1002/clc.20713 PMC6652867

[42] Mariscalco G, Engstrom KG, Ferrarese S, Cozzi G, Bruno VD, et al (2006) Relationship between atrial histopathology and atrial fibrillation after coronary bypass surgery. J Thorac Cardiovasc Surg 131: 1364–1372. 16733171 10.1016/j.jtcvs.2006.01.040

[43] Lei B, Hitomi H, Mori T, Nagai Y, Deguchi K, et al (2011) Effect of efonidipine on TGF-beta1-induced cardiac fibrosis through Smad2-dependent pathway in rat cardiac fibroblasts. J Pharmacol Sci 117: 98–105. 21897055 10.1254/jphs.11065fp PMC3230079

[44] Razani B, Zhang XL, Bitzer M, von Gersdorff G, Bottinger EP, et al (2001) Caveolin-1 regulates transforming growth factor (TGF)-beta/SMAD signaling through an interaction with the TGF-beta type I receptor. J Biol Chem 276: 6727–6738. 11102446 10.1074/jbc.M008340200

[45] Moe GW, Laurent G, Doumanovskaia L, Konig A, Hu X, et al (2008) Matrix metalloproteinase inhibition attenuates atrial remodeling and vulnerability to atrial fibrillation in a canine model of heart failure. J Card Fail 14: 768–776. 18995182 10.1016/j.cardfail.2008.07.229

[46] Seto SW, Krishna SM, Yu H, Liu D, Khosla S, et al (2013) Impaired acetylcholine-induced endothelium-dependent aortic relaxation by caveolin-1 in angiotensin II-infused apolipoprotein-E (ApoE-/-) knockout mice. PLoS One 8: e58481. 23469284 10.1371/journal.pone.0058481 PMC3587590

[47] Ma F, Li Y, Jia L, Han Y, Cheng J, et al (2012) Macrophage-stimulated cardiac fibroblast production of IL-6 is essential for TGF beta/Smad activation and cardiac fibrosis induced by angiotensin II. PLoS One 7: e35144. 22574112 10.1371/journal.pone.0035144 PMC3344835

