# Correction: Role of Caveolin-1 in Atrial Fibrillation as an Anti-Fibrotic Signaling Molecule in Human Atrial Fibroblasts

**DOI:** 10.1371/journal.pone.0224190

**Published:** 2019-10-18

**Authors:** Shao-lei Yi, Xiao-jun Liu, Jing-quan Zhong, Yun Zhang

There is an error in Fig 5, which incorrectly shows duplicate concentration series data in both panels 5A and 5B. The authors provide a revised Fig 5 which includes a corrected panel 5A showing time series data here.

**Fig 5:**
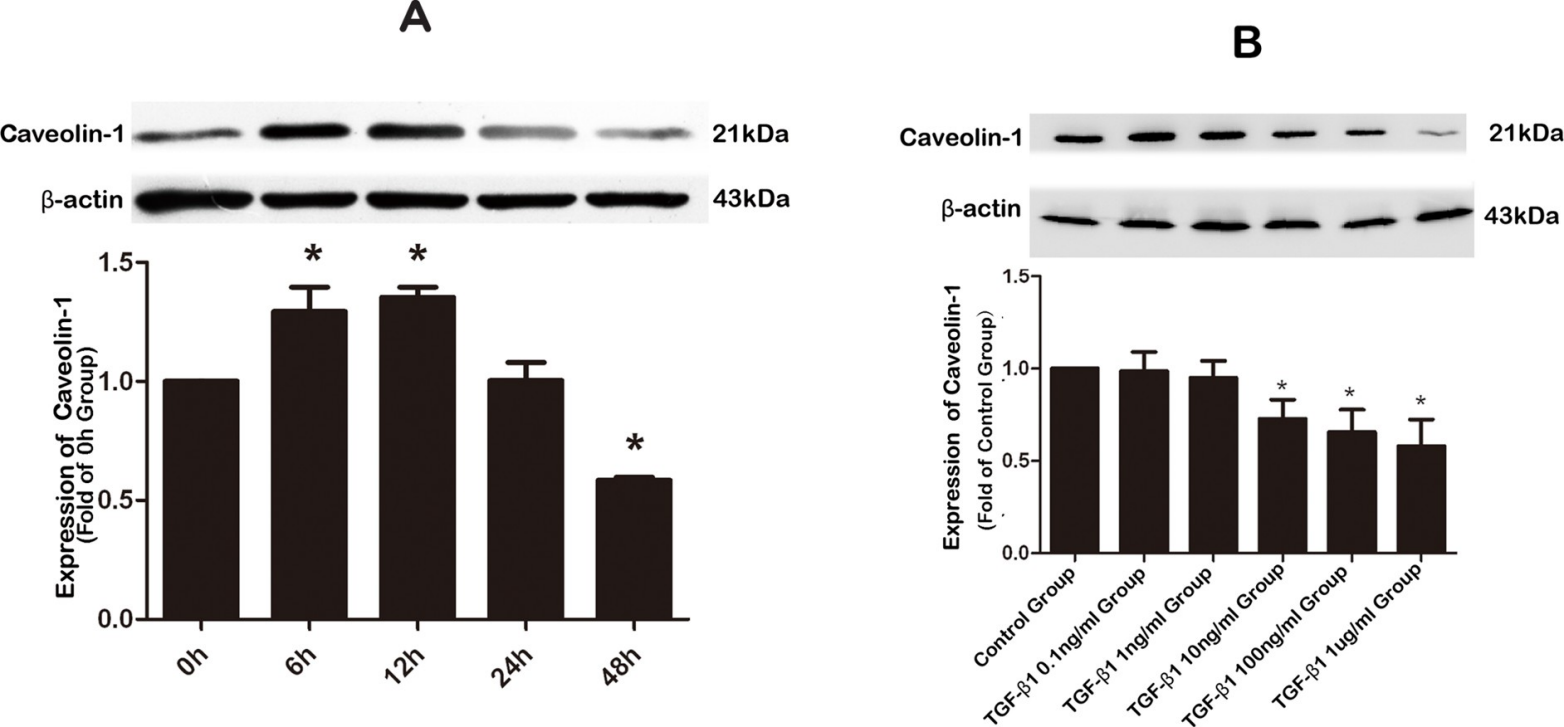
Effects of TGF-β1 on the expression of Cav-1 in human atrial fibroblasts (HAF). **A**, Time-dependent alterations of Cav-1 protein levels assessed by Western blotting in HAFs incubated with TGF-β1 of 100 ng/ml for varying periods (0, 6, 12, 24, and 48 h). Note that TGF-β1 induced bi-phasic changes of Cav-1 protein expression with initial upregulation followed by subsequent downregulation. Control HAFs were mock-treated. **B**, Concentration-dependent effects of TGF-β1 on Cav-1 protein levels assessed by Western blot analysis in HAFs exposed to varying concentrations of TGF-β1 (0, 0.1, 1, 10, 100, and 1000 ng/ml) for 48 h. *P<0.05 vs. Control; n = 3 per group with each measurement conducted in triplicate.

The available underlying dataset for Fig 5 is included as Supporting Information. The images for Fig 5A were recorded using Kodak film. The images for Fig 5B were recorded using the automatic gel imaging system (Syngene). To prepare the figures, raw images were converted from color to grayscale, and adjustments were made to the brightness, contrast, angle, and size of the image.

For the western blot experiments, after sodium dodecyl sulfate polyacrylamide gel electrophoresis, the whole gel was divided according to the molecular weight of the target protein. From here, the strips of gel containing different proteins were processed in parallel. Therefore, all figures in the article were assembled using separate blots for each target protein row, but this was not clearly represented in the figures due to the close placement of the images.

During the experimental process, at least three batches of samples were tested, and each batch of sample was tested in triplicate. When analyzing results, the normalization of protein expression data was conducted using the corresponding bands of β-actin from the same lanes of the same gel, with the exception of the p-Smad experiments shown in Figures 7 and 9. During the experimental process for p-Smad2, p-Smad3 and t-Smad, the identical amounts of the same samples were added to three groups of wells within the same gel. The ratio of p-Smad and total Smad was analysed using data from the corresponding sample in each group within the same gel. An illustration of the western blot methodology is included as Supporting Information.

In Fig 5B, there is a broad curvature to the β-actin row of bands, which is not present in the corresponding Caveolin-1 row. The authors confirm that the Caveolin-1 bands and the β-actin bands shown in Fig 5B are taken from the same sample run in the same lanes on the same gel. The difference in curvature may be caused by the positioning of individual gel/membrane strips during processing.

The original materials underlying the other figures are no longer available. The representative rows of bands shown within each panel in the figures are taken from the triplicate assessment of the same samples, either from the same gel or loaded onto a different gel in the same order, at the same time.

The original underlying data files for the other figures in the article are no longer available.

There is an update to the corresponding author’s contact information for this article. Jing-quan Zhong’s current email address is: 18560086597@163.com.

## Supporting information

S1 File Fig 5A actin blot. (TIF)

S2 File Fig 5A caveolin-1 blot. (TIF)

S3 File Fig 5B actin blot. (TIF)

S4 File Fig 5B caveolin-1 blot. (TIF)

S5 File Data points for Fig 5A. (DOC)

S6 File Data points for Fig 5B. (DOC)

S7 File Western blot methodology. (DOC)
