# Supplementary figures and images for: Role of Caveolin-1 in Atrial Fibrillation as an Anti-Fibrotic Signaling Molecule in Human Atrial Fibroblasts
Source: PLoS One. 2014 Jan 14;9(1):e85144. doi: 10.1371/journal.pone.0085144 (PMC3891766; doi:10.1371/journal.pone.0085144)

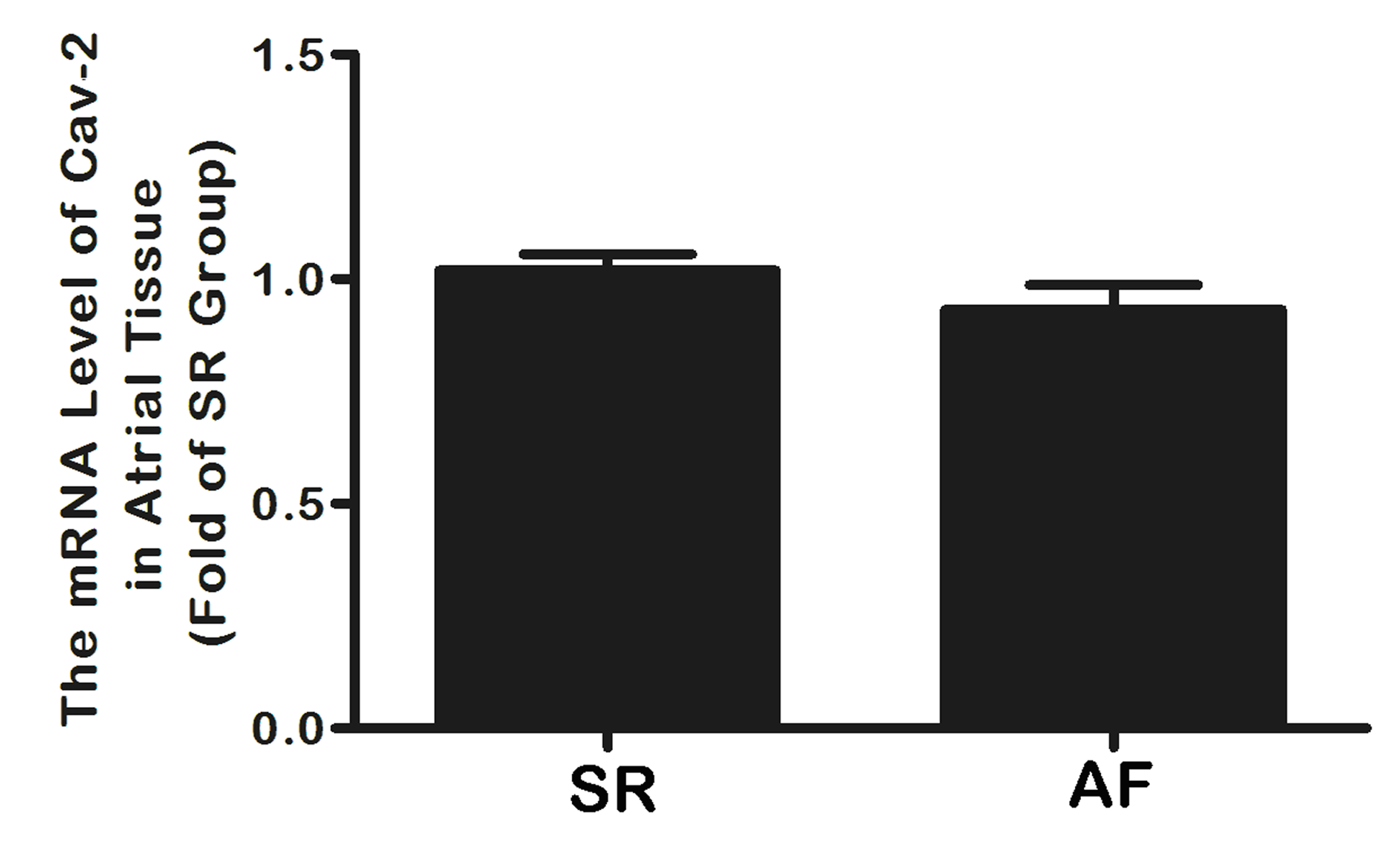

Supplement: Figure S1 — Comparison of atrial Cav-2 mRNA levels between AF and SR subjects, as determined by real-time RT-PCR (qRT-PCR). P = 0.325, n = 6. Note that the gene expression of Cav-2 showed no significant difference between groups. Cav-2, caveolin-2; SR, sinus rhythm; AF, atrial fibrillation. (TIF) [file pone.0085144.s001.tif]

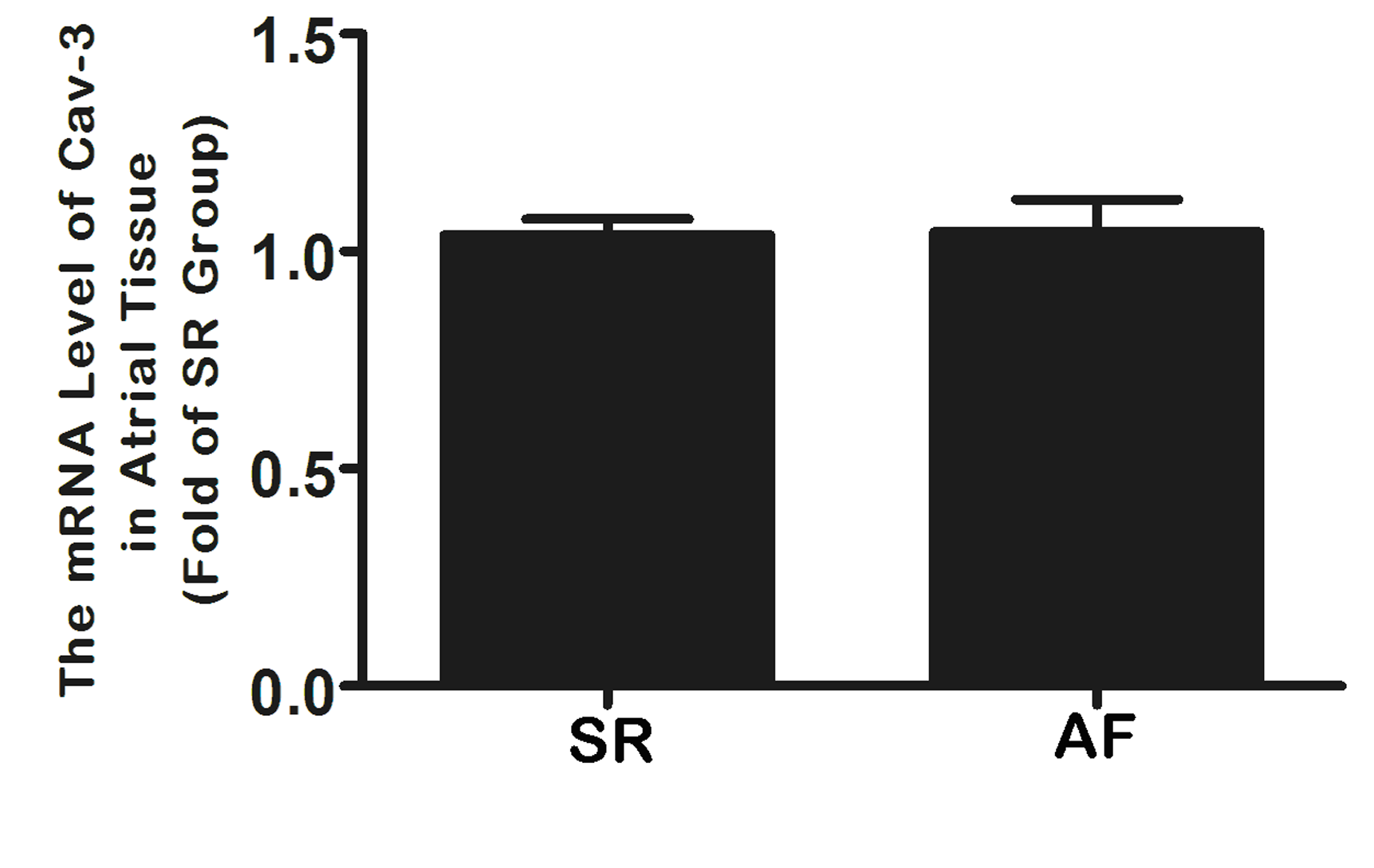

Supplement: Figure S2 — Comparison of atrial Cav-3 mRNA levels between AF and SR subjects, as determined by real-time RT-PCR (qRT-PCR). P = 0.943, n = 4. Note that the gene expression of Cav-3 showed no significant difference between groups. Cav-3, caveolin-3; SR, sinus rhythm; AF, atrial fibrillation. (TIF) [file pone.0085144.s002.tif]
