# Supplementary material for: Role of Caveolin-1 in Atrial Fibrillation as an Anti-Fibrotic Signaling Molecule in Human Atrial Fibroblasts
Source: PLoS One. 2014 Jan 14;9(1):e85144. doi: 10.1371/journal.pone.0085144 (PMC3891766; doi:10.1371/journal.pone.0085144)
Supplement: File S1 — The protocols of western blot, qRT-PCR and Masson Staining. (DOC) [file pone.0085144.s003.doc]

**qRT-PCR.**

**Total RNA Isolation and Real-time quantitative RT-PCR**

Total RNA was isolated from each atrial tissue sample and cell cultures with TRIzol reagent (Invitrogen) using the manufacturer’s protocol. RNA amount was quantitated by a spectrophotometer (Bio-Rad Laboratories Inc), and the purity was determined from the A260/A280 ratio (at or above 1.80, to 2.00). RNA was then reverse transcribed to cDNA with RevertAid^TM^ First Strand cDNA Synthesis Kit (Fermentas) according to the manufacturer’s instructions, which was used as a template in the subsequent PCR analyses. Real-time PCR was performed using SsoFastTM EvaGreen Supermix (Bio-Rad Laboratories Inc) on a Bio-Rad iCycler iQ5 real-time PCR system (Bio-Rad, Hercules, CA). The human Caveolin-1, Caveolin-2, Caveolin-3, MMP-2, MMP-9 forward and reverse primers were: Caveolin-1: 5’-AATACTGGTTTTACCGCTTGCT-3’ and 5’-CATGGTACAACTGCCCAGATG-3’; Caveolin-2: 5’-TCCCCACCGGCTCAAC -3’ and 5’-GTCACCGGCTCTGCGAT-3’; Caveolin-3: 5’-GGTGCCATGCATTAAGAGCTA -3’ and 5’-TGCGGATGCAGAGTGAGTAG-3’; MMP-2: 5′-CCCACTGAGGAGTCCAACAT-3′ and 5′-CATTTACACGTCTGCGGATCT-3′; MMP9: 5′-TCCCTGGAGACCTGAGAACC-3′ and 5′-GGCAAGTCTTCCGAGTAGTTT-3′; Collagen I: 5′-ATGTCCACCGAGGCCTCCCAGAAC-3′ and 3′- CCCAGGCTCCGGTGTGACTCGTG-5′; Collagen III: 5′- CCTGGTCCTTGCTGTGGTGGTGT-3′ and 3′- GCAGTTTCTAGCGGGGTTTTTACG-5′. Primers were used at a concentration of 500nM in each reaction. Cycling conditions were set as follows: 95℃ for 30 seconds, 40 cycles of 95℃ for 5 seconds and 60℃ for 10 seconds. Data from the reaction were collected and analyzed by Bio-Rad iQ5 Optical System Software (Bio-Rad Laboratories Inc) using a standard curve. Relative quantitations of gene expression were normalized toβ-actin. The results of real-time PCR were expressed as 2–ΔΔCT values.

**Western Blot Analysis**

Proteins were extracted from atrial tissue samples and treated cells on ice with 200μL lysis buffer and 2μL phenylmethylsulfonyl fluoride. Samples were incubated on ice for 30 minutes, followed by 10 minutes of centrifugation at 14,000 revolutions per minute at 4℃. The supernatants were collected and the protein concentration was assessed by BCA Protein Assay Kit (Pierce) according to manufacturer’s instructions. Proteins were heated at 99℃ for 5 minutes before loading, separated by sodium dodecyl sulfate polyacrylamide gel electrophoresis (SD-PAGE) (10% polyacrylamide), and then transferred to polyvinylidene difluoride membranes (Millipore). After blocked with 5% non-fat dry milk/TBS with 0.1% (Vol/Vol) Tween 20 for 2 hours, specific proteins were stained using the following primary antibodies: Cav-1 antibody (1:1,000 dilution), rabbit polyclonal to TGF-β1 (1:100 dilution), collagen I(1:1,000 dilution), collagen Ⅲ(1:1,000 dilution), MMP-1(1:800 dilution), MMP-2(1:800 dilution), MMP-9(1:800 dilution). Monoclonal antibody against β-actin was used in every experiment for the internal control. Horseradish peroxidase conjugated anti-mouse or rabbit IgG was used as a secondary antibody. Antibody-bound proteins were visualized by ECL. Densitometric evaluation of the detected bands was performed using [Adobe Photoshop CS5](http://baike.baidu.com/view/3505182.htm) (Adobe Systems Inc, CA, USA).

**Masson Staining**

Atrial tissue samples were fixed in 10% neutralized formalin for 24 hours and processed for paraffin embedding. Paraffin blocks were cut into 5-mm sections and mounted to slides. Sections of atrial tissue were stained with Masson-trichrome to assess the degree of fibrosis. At least 6 fields at 200-fold magnification were captured in all the samples. The percentages of fibrotic area in atrial tissue samples were determined by Image-Pro Plus 6.0 software (Media Cybernetics Inc, Beijing, China).
