# Supplementary figures and images for: Correction: Role of Caveolin-1 in Atrial Fibrillation as an Anti-Fibrotic Signaling Molecule in Human Atrial Fibroblasts
Source: PLoS One. 2019 Oct 18;14(10):e0224190. doi: 10.1371/journal.pone.0224190 (PMC6799895; doi:10.1371/journal.pone.0224190)

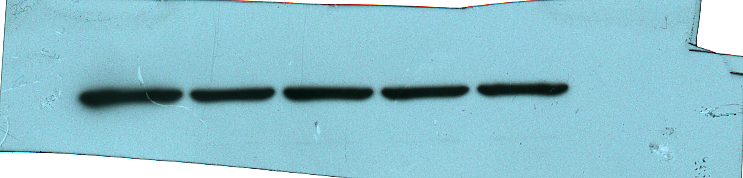

Supplement: S1 File — (TIF) [file pone.0224190.s001.tif]

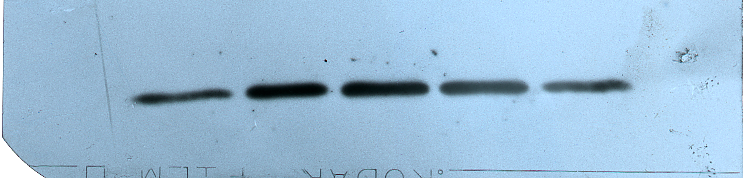

Supplement: S2 File — (TIF) [file pone.0224190.s002.tif]
