# Supplementary material for: Correction: Role of Caveolin-1 in Atrial Fibrillation as an Anti-Fibrotic Signaling Molecule in Human Atrial Fibroblasts
Source: PLoS One. 2019 Oct 18;14(10):e0224190. doi: 10.1371/journal.pone.0224190 (PMC6799895; doi:10.1371/journal.pone.0224190)
Supplement: S5 File — (DOC) [file pone.0224190.s005.doc]

|  | Relative expression of caveolin-1( mean± SE) |
| --- | --- |
| 0h | 1±0 |
| 6h | 1.29257062±0.102738828 |
| 12h | 1.35266778±0.043173434 |
| 24h | 1.00343849±0.075327171 |
| 48h | 0.58391685±0.001321356 |

Individual-level data points for figure 5A
