# Supplementary material for: Correction: Role of Caveolin-1 in Atrial Fibrillation as an Anti-Fibrotic Signaling Molecule in Human Atrial Fibroblasts
Source: PLoS One. 2019 Oct 18;14(10):e0224190. doi: 10.1371/journal.pone.0224190 (PMC6799895; doi:10.1371/journal.pone.0224190)
Supplement: S6 File — (DOC) [file pone.0224190.s006.doc]

|  | Relative expression of caveolin-1( mean± SE) |
| --- | --- |
| Control group | 1.0000±0.00000 |
| TGF-β1 0.1ng/ml group | 0.9855±0.10342 |
| TGF-β1 1ng/ml group | 0.9490±0.09314 |
| TGF-β1 10ng/ml group | 0.6944±0.09687 |
| TGF-β1 100ng/ml group | 0.6555±0.12156 |
| TGF-β1 1ug/ml group | 0.5798±0.14510 |

Individual-level data points for figure 5B
