# Supplementary material for: Correction: Role of Caveolin-1 in Atrial Fibrillation as an Anti-Fibrotic Signaling Molecule in Human Atrial Fibroblasts
Source: PLoS One. 2019 Oct 18;14(10):e0224190. doi: 10.1371/journal.pone.0224190 (PMC6799895; doi:10.1371/journal.pone.0224190)
Supplement: S7 File — (DOC) [file pone.0224190.s007.doc]

During the experimental process, at least three batches of samples were tested and each batch of sample was tested in three different pieces of gels. When analyzing results, the normalization of target protein were conducted by comparing bands of β-actin coming from the same group of wells in the same piece of gel，as shown in picture 1.


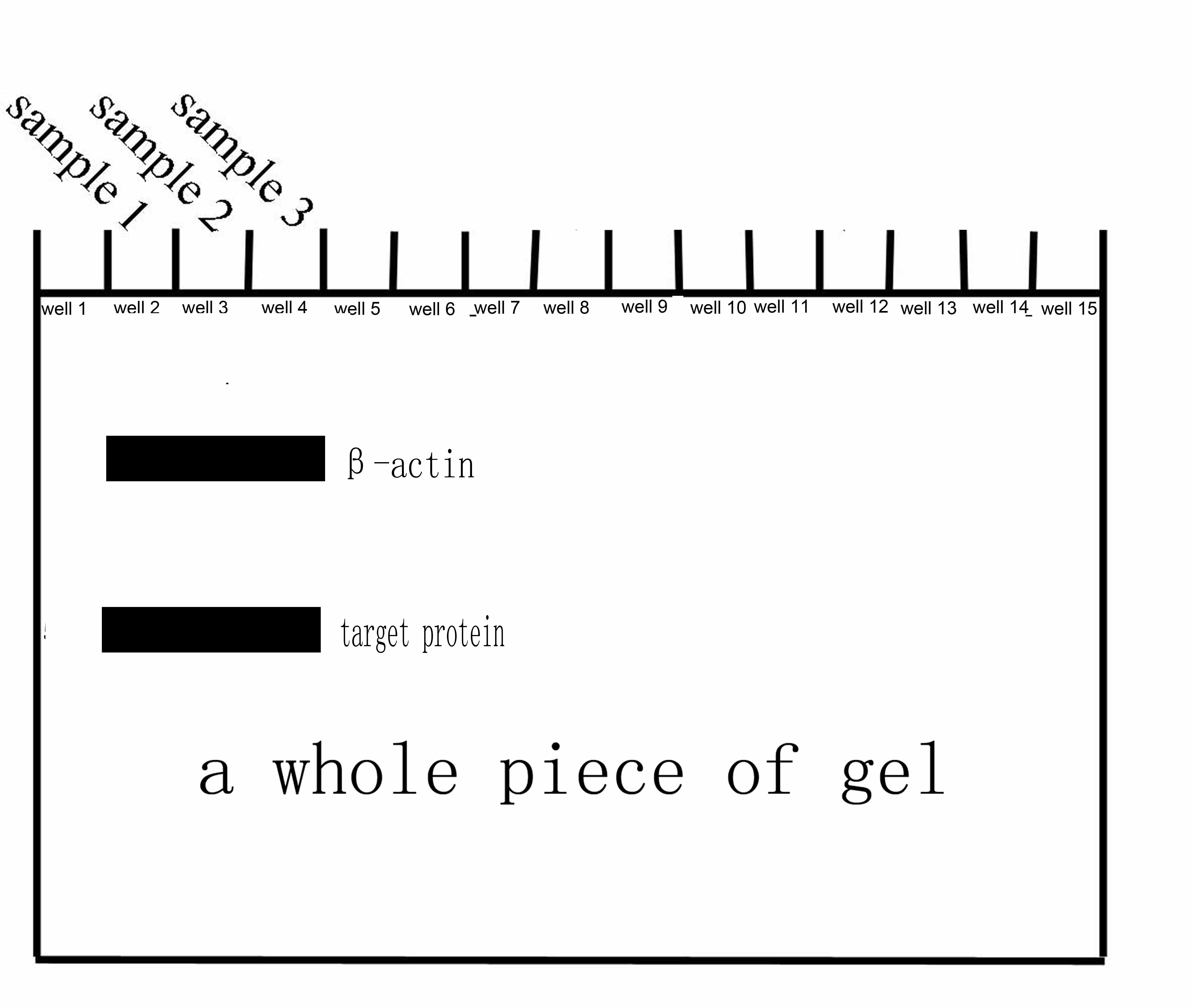


**Picture 1**

While for those proteins having similar molecular weight, such as p-Smad2, p-Smad3 and t-Smad, the identical amounts of the same samples were added to 3 groups of wells repeatedly in a gel in the same time (picture 2). When analyzing, the ratio of p-smad and total smad coming from different groups in the same piece of gel was used to reflect the activation of smads signaling pathway.


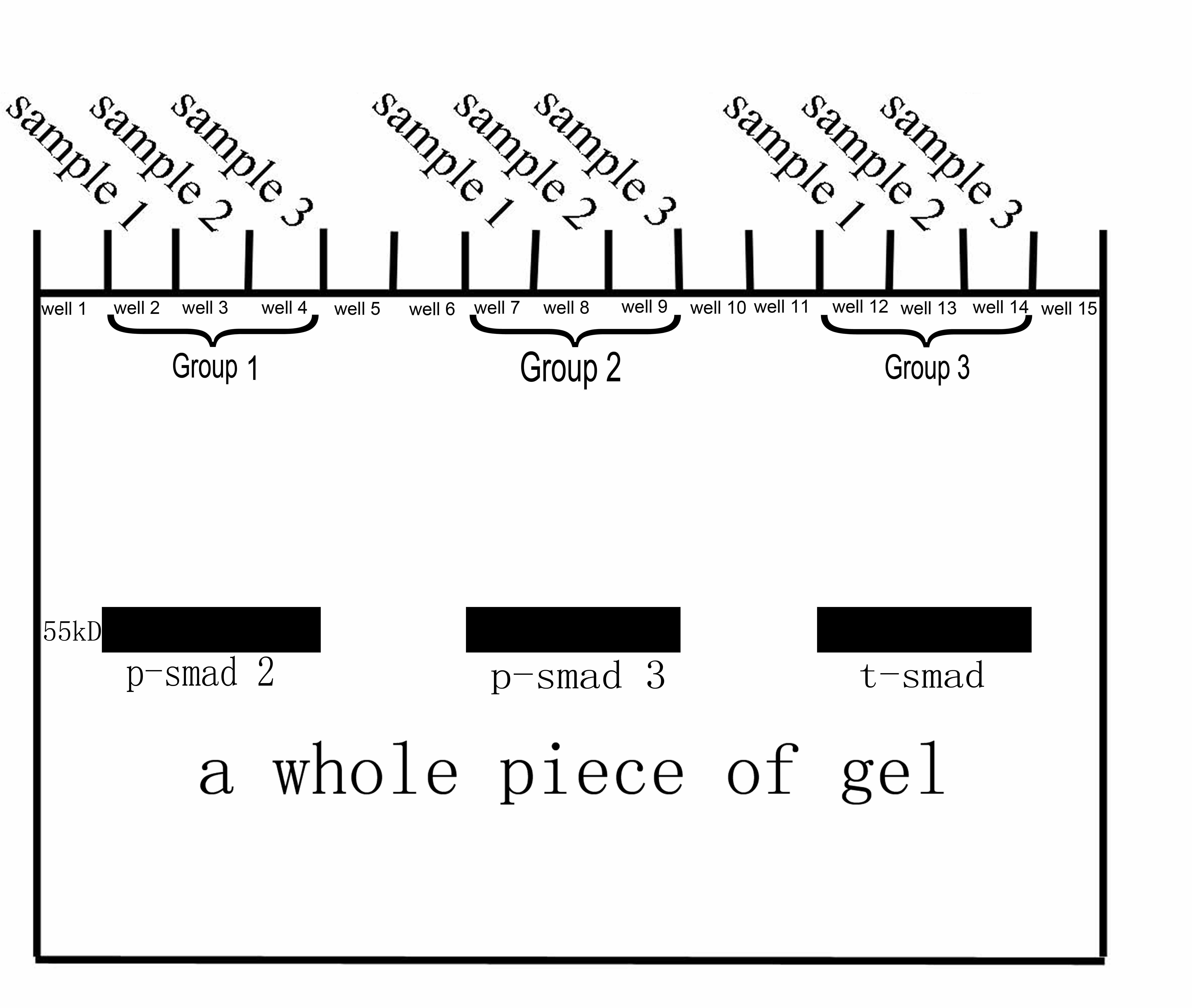


**Picture 2**
